# Impact of *CYP1A1* variants on the risk of acute lymphoblastic leukemia: evidence from an updated meta-analysis

**DOI:** 10.1007/s44313-024-00007-9

**Published:** 2024-03-04

**Authors:** Imen Frikha, Rim Frikha, Moez Medhaffer, Hanen Charfi, Fatma Turki, Moez Elloumi

**Affiliations:** 1 Faculty of Medicine of Sfax, University of Sfax, Sfax, Tunisia; 2 Department of Hematology, Hedi Chaker Hospital, Sfax, Tunisia; 3 Department of Medical Genetics, Hedi Chaker Hospital, Sfax, Tunisia

**Keywords:** Cytochrome, Acute lymphoblastic leukemia, Risk, Meta-analysis

## Abstract

**Objective:**

Our study aimed to investigate the association between cytochrome P450 1A1 (*CYP1A1*) polymorphisms (T3801C and A2455G) and acute lymphoblastic leukemia (ALL) risk, considering genetic models and ethnicity.

**Materials and methods:**

PubMed, Embase, Web of Knowledge, Scopus, and the Cochrane electronic databases were searched using combinations of keywords related to *CYP1A1* polymorphisms and the risk of ALL. Studies retrieved from the database searches underwent screening based on strict inclusion and exclusion criteria.

**Results:**

In total, 2822 cases and 4252 controls, as well as 1636 cases and 2674 controls of the C3801T and A2455G variants of CYP1A1, respectively, were included in this meta-analysis. The T3801C polymorphism of CYP1A1 significantly increases the risk of ALL, particularly those observed in Asian and Hispanic populations, independent of age. Similarly, the A2455G polymorphism of CYP1A1 plays a significant role in the susceptibility to ALL in all genetic models, except the heterozygous form. This association was observed mainly in mixed populations and in both children and adults (except in the heterozygous model).

**Conclusion:**

Our comprehensive analysis indicates that the T3801 and A2455G polymorphisms of *CYP1A1* may increase the risk of ALL depending on ethnicity. Therefore, both variants should be considered promising biomarkers for ALL risk. Further large-scale investigations are necessary to assess other factors, such as gene-gene or gene-environment interactions.

**Supplementary Information:**

The online version contains supplementary material available at 10.1007/s44313-024-00007-9.

## Introduction

Acute lymphoblastic leukemia (ALL) stands as the most common pediatric cancer, constituting approximately 30% of all childhood cancer cases. However, it also affects individuals of all ages. The malignant transformation and uncontrolled proliferation of abnormally differentiated, long-lived hematopoietic progenitor cells lead to a significant presence of circulating blasts and malignant cells, ultimately replacing normal marrow [1]. The causes of ALL remain unknown and likely involve interactions among the environment, hematopoietic development, and low-penetrance susceptibility loci [2]. This complexity arises from the intricate interplay between genetic predispositions and environmental factors [3]. Environmental chemicals necessitate metabolic activation by phase I xenobiotic-metabolizing enzymes, such as cytochrome P450, for conversion into carcinogens [4]. Polymorphisms in genes encoding xenobiotic-metabolizing enzymes can modify the expression or activity of these enzymes, thereby influencing the risk of exposure-related cancers [5–7]. Additionally, the association with cancer risk may be age-dependent due to distinct developmental patterns exhibited by many xenobiotic-metabolizing enzymes, such as cytochrome P450 1A1 (CYP1A1) [8].

The gene for CYP1A1, which encodes a significant cytochrome P450 enzyme, harbors two crucial single-nucleotide polymorphisms: CYP1A1*2A (rs4646903) and CYP1A12C (rs1048943) [6]. CYP1A12A involves the substitution of T with C in the 3′ untranslated regions, also known as T3801C, m1, or rs4646903. This single nucleotide polymorphism (SNP) is localized on chromosome 15q22 [9]. CYP1A1*2C results from an A-to-G transition, leading to an isoleucine/valine substitution in exon 7, also referred to as A2455G, m2 allele, or rs1048943 [10]. Numerous studies have investigated the relationship between CYP1A1 polymorphisms and leukemia risk, but the results have been inconsistent [11–14]. Therefore, we conducted a meta-analysis of all eligible studies, including ALL in both adults and children, to clarify the associations of *CYP1A1* polymorphisms (T3801C and A2455G) with leukemia risk based on genetic models and ethnicity.

## Materials and methods

### Literature search

A comprehensive search was conducted to investigate the association between CYP1A1 polymorphisms and the risk of ALL published before February 2020, utilizing the PubMed electronic database. The following combined MeSH terms were employed: “CYP1A1,” “polymorphisms,” and “Acute lymphoblastic leukemia” OR “Acute lymphocytic leukemia.” The search was conducted without any language restrictions. Additionally, the reference lists of identified articles were manually searched for additional relevant publications. Studies were included in the current meta-analysis if they assessed the association between two *CYP1A1* polymorphisms (T3801C and A2455G) and ALL.

### Study selection

Publications were eligible for the meta-analysis if they met the following inclusion criteria: (1) case–control studies; (2) studies determining the distribution of CYP1A1 variants (2A (T > C) rs4646903 and CYP1A12B/2C (A2455G) rs1048943) in patients and controls, evaluating polymorphisms and their association with susceptibility to ALL; and (3) sufficient genotype frequency data available for both case and control groups.

The major exclusion criteria were as follows: (1) patients with other leukemias (such as AML, and CML); and (2) data duplicated from previous publications.

### Data extraction

To minimize bias in the selected studies, two observers independently extracted information from each study and resolved all disagreements through discussion. The following information was extracted from each study: (1) the first author, (2) year of publication, (3) country of the study population, (4) ethnicity, (5) CYP1A1 gene variants, and (6) age. Additionally, we checked the distribution of genotypes in controls using Hardy–Weinberg equilibrium (HWE).

### Statistical analysis

Meta-analysis was conducted using the Metagenyo software [15]. It is an easy-to-use web application that implements a comprehensive meta-analysis workflow for Genetic Association Studies (GAS). Various genetic comparison models were employed to assess the association between *CYP1A1* polymorphisms and the risk of ALL, including allele contrast, recessive, dominant, homozygous, and heterozygous models. The genotype distribution was assessed for HWE to check the study quality; p (HWE) < 0.05 showed statistical significant [15]. Odds ratios (OR) and 95% confidence intervals (CI) were calculated using the genotype distribution data. These association test results assessed the strength of the association between CYP1A1 polymorphisms (T3801C and A2455G) and the risk of ALL.

Heterogeneity among studies was evaluated using Cochran’s Q-statistic (*p* < 0.05) and the I^2^ test, representing the percentage of total variation across studies ranging from 0 to 100%. I^2^ ≤ 25%, 25% < I^2^ < 50%, and I^2^ > 50% were interpreted as low, moderate, and a high degree of heterogeneity, respectively [16]. A random-effects model was applied if there was significant heterogeneity (*p* < 0.05, I^2^ > 50%); otherwise, a fixed-effects model was employed [17].

*A funnel plot* and graphical tests were used to assess publication bias. Therefore, the asymmetry of the funnel plot suggests possible publication bias. *Egger’s test* was used; *p* < 0,05 is considered a potential statistical publication bias [16]. *Forest plots* were provided by meta-analysis to estimate the global results of all studies. Egger’s test and funnel plots were used to identify statistically significant publication bias (*p* < 0.05) [16]. Sensitivity analyses were conducted to examine whether the individual study influenced the pooled results.

## Results

### Search published reports

**Fig. 1:**
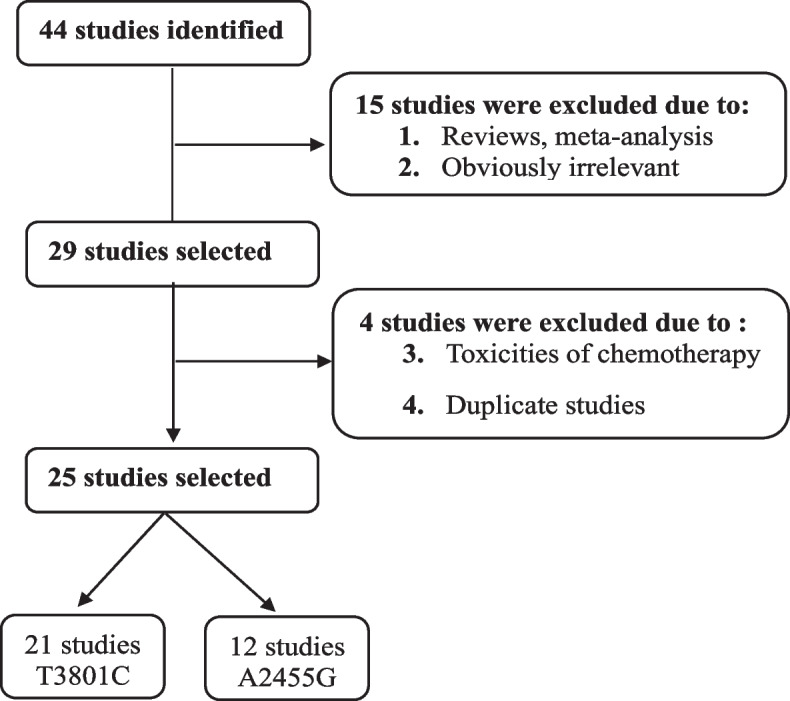
Flow diagram for article identification and exclusion

A flowchart illustrating the study selection process is presented in Fig. 1. Articles were identified using various combinations of keywords listed in the Methods section, focusing on the relationship between CYP1A1 SNPs and the risk of ALL. Seven of these articles were reviews or meta-analyses. Consequently, only 25 studies were included in this meta-analysis.

### Study characteristics

The characteristics of the 25 articles included in the meta-analysis are outlined in Table 1. These studies were conducted in diverse populations with varying ethnicities, including Asian, African, Caucasian, Hispanic, and mixed-ethnic groups.

**Table 1:** Characteristics of included studies

First author	Year of publication	Ethnicity (Country)	Polymorphism Study	Age group	Number	
					Cases	Controls
***Krajinovic*** [18]	1999	Caucasian (Canada)	T3801C, A2455G	Child	517	893
***Gao*** [19]	2003	Asian (China)	T3801C, A2455G	Child	156	225
***Balta*** [20]	2003	Mixed (Turky)	T3801C	Child	105	145
***Joseph*** [21]	2004	Asian (India)	T3801C, A2455G	Child	236	236
***Canalle*** [22]	2004	Mixed (Brazil)	T3801C	Child	113	221
***Gallegos-Arreole*** [23]	2004	Mixed (Mexican)	A2455G	Adult	136	136
***Selvin*** [24]	2004	Mixed (California)	A2455G	Child	175	175
***Clavel*** [25]	2005	Mixed (France)	T3801C	Child	190	105
***Liu QX*** [26]	2005	Asian (China)	T3801C	Adult	112	179
***Pakakasama*** [27]	2005	Asian (Thailand)	T3801C	Child	91	320
***Aydin-Sayitoglu*** [28]	2006	Caucasian (Turky)	T3801C	Adult	36	140
***Aydin-Sayitoglu*** [28]	2006	Caucasian (Turky)	T3801C	Child	119	140
***Bolufer*** [13]	2007	Caucasian (Spain)	T3801C	Mixt	92	403
***Gallegos-Arreola*** [29]	2008	Hispanic (Mexican)	T3801C	Adult	210	228
***Chen*** [30]	2008	Asian (China)	T3801C	Mixt	120	204
***Lee*** [31]	2009	Asian (Korea)	T3801C, A2455G	Child	207	321
***Yamaguti*** [14]	2010	Caucasian (Brazil)	T3801C, A2455G	Mixt	198	198
***Swinney*** [32]	2011	Hispanic (USA)	T3801C, A2455G	Child	302	814
***Swinney*** [32]	2011	Caucasian (USA)	A2455G	Child	171	437
***Swinney*** [32]	2011	Caucasian (USA)	A2455G	Child	71	204
***Suneetha*** [33]	2011	Asian (India)	T3801C	Child	91	150
***Razmkhah*** [34]	2011	Mixed (Iran)	A2455G	Child	85	94
***Bonaventure*** [35]	2012	Caucasian (France)	T3801C	Child	430	548
***Agha*** [36]	2013	African (Egypt)	T3801C, A2455G	Child	558	600
***Ouerhani*** [37]	2013	African (Tunisia)	T3801C	Mixt	100	106
***Nida*** [11]	2017	Asian (India)	T3801C	Child	200	200

### Variants allele frequency

The allele frequencies of each variant of the CYP1A1 genotypes in the case and control groups are summarized in Tables 2, 3, 4 and 5. For the *CYP1A1 T3801C* polymorphism, 2822 patients and 4252 controls were enrolled in these studies. In the case group, the C allele frequencies ranged from 6 to 57% (mean = 20.6%; 95% CI [18.3–32.8]). The frequencies of the C allele varied by ethnicity: (22–51%), (7–21%), (11–20%), (21–60%), and (6–11%) in Asian, Caucasian, mixed, Hispanic, and African populations, respectively. In the control group of the T3801C polymorphism, T allele frequencies ranged from 15.4% to 27% (mean = 25%) (*p* = 0.3).

**Table 2:** Results of meta-analysis for the CYP1A1 gene T3801C polymorphisms in ALL according to the ethnicity

**Model**	**Ethnicity**	**Number of studies**	**Test of association**			**Test of heterogeneity**			**Publication bias**
			**OR**	**95% CI**	***p*-val**	**Model**	***p*-val**	**I**^**2**^	***p*-val (Egger's test)**
**Allelecontrast** ***(C vs. T)***	Overall	21	1,31	[1.08; 1.58]	0,00*	Random	0,00*	75,46%	0,21
	Asian	8	1,36	[1.07; 1.71]	0,00*	Random	0,00*	64,87%	0,07
	African	2	1,48	[0.92; 2.37]	0,10	Fixed	0,16	49,35%	NA
	Caucasian	6	1,10	[0.91; 1.33]	0,31	Fixed	0,14	38,9%	0,47
	Hispanic	2	2,22	[0.91; 5.43]	0,07	Random	0,00*	91,94%	NA
	Mixed	3	1,05	[0.80; 1.38]	0,68	Fixed	0,66	0%	0,13
**Recessive model** ***(CC vs. CT+TT)***	Overall	21	1,36	[0.86; 2.16]	0,18	Random	0,00*	72,21%	0,68
	Asian	8	1,01	[0.78; 1.31]	0,92	Fixed	0,16	33,11%	0,07
	African	2	2,21	[0.47; 10.44]	0,31	Fixed	0,50	0%	NA
	Caucasian	6	0,63	[0.30; 1.33]	0,23	Fixed	0,94	0%	0,51
	Hispanic	2	4,91	[1.61; 14.93]	0,00*	Random	0,01*	82,35%	NA
	Mixed	3	1,44	[0.18; 11.56]	0,72	Random	0,05	66,3%	0,77
**Dominant model** ***(CC+CT vs. TT)***	Overall	21	1,40	[1.17; 1.68]	0,00*	Random	0,00*	56,88%	0,56
	Asian	8	1,67	[1.30; 2.15]	6,14E-05*	Random	0,09	42,96%	0,44
	African	2	1,43	[0.86; 2.37]	0,16	Fixed	0,18	41,92%	NA
	Caucasian	6	1,17	[0.95; 1.44]	0,12	Fixed	0,10	45,29%	0,66
	Hispanic	2	1,80	[0.71; 4.59]	0,21	Random	0,01*	84,68%	NA
	Mixed	3	1,02	[0.75; 1.39]	0,85	Fixed	0,63	0%	0,59
**Homozygous** ***(CC vs.TT)***	Overall	21	1,59	[0.99; 2.53]	0,05	Random	0,00*	68,49%	0,47
	Asian	8	1,41	[0.92; 2.15]	0,10	Random	0,06	47,23%	0,11
	African	2	2,27	[0.48; 10.72]	0,29	Fixed	0,47	0%	NA
	Caucasian	6	0,67	[0.32; 1.41]	0,29	Fixed	0,96	0%	0,42
	Hispanic	2	5,02	[1.26; 20.00]	0,02*	Random	0,00*	86,21%	NA
	Mixed	3	1,46	[0.19; 10.86]	0,70	Random	0,06	63,5%	0,77
**Heterozygous** ***(CT vs. TT)***	Overall	21	1,38	[1.17; 1.64]	0,00*	Random	0,01*	44,66%	0,55
	Asian	8	1,83	[1.48; 2.25]	1,16E-08*	Fixed	0,29	17,31%	0,25
	African	2	1,34	[0.79; 2.27]	0,26	Fixed	0,23	27,98%	NA
	Caucasian	6	1,22	[0.89; 1.68]	0,20	Random	0,09	47,18%	0,83
	Hispanic	2	1,18	[0.81; 1.71]	0,37	Fixed	0,18	43,82%	NA
	Mixed	3	1	[0.73; 1.37]	0,99	Fixed	0,34	05,98%	0,93

*NA* Not available, *OR* Odds ratio

*significance (*p* < 0.05)

**Table 3:** Results of meta-analysis for the CYP1A1 gene T3801C polymorphisms in ALL according to the Age

**Model**	**Age**	**Number of studies**	**Test of association**			**Test of heterogeneity**			**Publication bias**
			**OR**	**95% CI**	***p*-val**	**Model**	***p*-val**	**I**^**2**^	***p*-val (Egger's test)**
**Allelecontrast** **(C vsT)**	Overall	21	1,31	[1.08; 1.58]	0,00*	Random	0,00*	75,46%	0,21
	Child	14	1,27	[1.08; 1.49]	0,00*	Random	0,02*	48,67%	0,20
	Mixt	4	1,14	[0.76; 1.72]	0,51	Random	0,05	60,27%	0,95
	Adult	3	1,57	[0.67; 3.68]	0,29	Random	0,00*	92,11%	0,50
**Recessive model** **(CC vs CT+TT)**	Overall	21	1,36	[0.86; 2.16]	0,18	Random	0,00*	72,21%	0,68
	Child	14	1,26	[0.81; 1.96]	0,29	Random	0,03*	46,09%	0,57
	Mixt	4	1,03	[0.63; 1.69]	0,88	Fixed	0,51	0	0,93
	Adult	3	1,88	[0.28; 12.56]	0,51	Random	0,00*	93,74%	0,85
**Dominant model ** **(CC+CT vs TT)**	Overall	21	1,40	[1.17; 1.68]	0,00*	Random	0,00*	56,88%	0,56
	Child	14	1,35	[1.12; 1.62]	0,00*	Random	0,06	40,07%	0,36
	Mixt	4	1,25	[0.76; 2.05]	0,37	Random	0,05	61,45%	0,99
	Adult	3	1,85	[1.00; 3.41]	0,04	Random	0,03*	71,08%	0,00
**Homozygous** **(CC vs TT)**	Overall	21	1,59	[0.99; 2.53]	0,05	Random	0,00*	68,49%	0,47
	Child	14	1,41	[0.90; 2.20]	0,12	Random	0,04*	42,59%	0,51
	Mixt	4	1,31	[0.75; 2.28]	0,33	Fixed	0,44	0	0,91
	Adult	3	2,56	[0.48; 13.57]	0,26	Random	0,00*	89,84%	0,72
**Heterozygous** **(CT vs TT)**	Overall	21	1,38	[1.17; 1.64]	0,00*	Random	0,01*	44,66%	0,55
	Child	14	1,38	[1.12; 1.69]	0,00*	Random	0,03*	45,18%	0,28
	Mixt	4	1,27	[0.78; 2.07]	0,32	Random	0,07	57,24%	0,97
	Adult	3	1,59	[1.14; 2.21]	0,00*	Fixed	0,11	54,17%	0,74

*NA* Not available, *OR* Odds ratio

*significance (*p* < 0.05)

**Table 4:** Results of meta-analysis for the CYP1A1 gene A2455G polymorphisms and ALL risk according to the ethnicity

**Model**	**Ethnicity**	**Number of studies**	**Test of association**			**Test of Heterogeneity**			**Publication bias**
			**OR**	**95% CI**	***p*-val**	**Model**	***p*-val**	**I**^**2**^	***p*-val (Egger's test)**
**Allelecontrast** ***(G vs. A)***	Overall	12	1,25	[1.09; 1.42]	0,00*	Fixed	0,14	30,92%	0,76
	Caucasian	5	1,21	[0.95; 1.54]	0,11	Fixed	0,81	0,00%	0,39
	Asian	3	1,36	[0.80; 2.31]	0,25	Random	0,01*	76,95%	0,11
	Mixed	4	1,26	[1.03; 1.53]	0,02*	Fixed	0,13	46,28%	0,69
**Recessive model ** ***(GG vs GA+AA)***	Overall	11	1,94	[1.39; 2.71]	0,00*	Fixed	0,79	0,00%	0,58
	Caucasian	5	2,03	[1.08; 3.83]	0,02*	Fixed	0,78	0,00%	0,76
	Asian	3	1,51	[0.81; 2.78]	0,18	Fixed	0,32	10,13%	0,45
	Mixed	3	2,25	[1.35; 3.73]	0,00*	Fixed	0,52	0,00%	0,46
**Dominant model ** ***(GG+GA vs AA)***	Overall	12	1,19	[1.01; 1.39]	0,03	Fixed	0,19	25,08%	0,77
	Caucasian	5	1,13	[0.85; 1.50]	0,37	Fixed	0,72	0,00%	0,54
	Asian	3	1,39	[0.75; 2.58]	0,28	Random	0,02*	71,87%	0,46
	Mixed	4	1,15	[0.91; 1.46]	0,23	Fixed	0,18	38,39%	0,94
**Homozygous** ***(GG vs AA)***	Overall	11	2,02	[1.43; 2.85]	0,00*	Fixed	0,69	0,00%	0,65
	Caucasian	5	1,92	[0.99; 3.70]	0,05	Fixed	0,86	0,00%	0,94
	Asian	3	1,67	[0.88; 3.17]	0,11	Fixed	0,22	33,47%	0,46
	Mixed	3	2,37	[1.41; 3.97]	0,00*	Fixed	0,32	11,46%	0,60
**Heterozygous** ***(GA vs AA)***	Overall	12	1,09	[0.92; 1.29]	0,3	Fixed	0,31	13,5%	0,54
	Caucasian	5	1,05	[0.78; 1.42]	0,72	Fixed	0,51	0,00%	0,64
	Asian	3	1,31	[0.75; 2.27]	0,33	Random	0,07	61,24%	0,35
	Mixed	4	1,03	[0.80; 1.32]	0,79	Fixed	0,33	11,26%	0,77

*NA* Not available, *OR* Odds ratio

*significance (*p* < 0.05)

Similarly, for the CYP1A1 A2455G polymorphism, 1636 cases and 2674 controls were included in these studies. In the case group, G allele frequencies varied from 4 to 41% (mean = 16.3%; 95% CI [10.1–23]). In the Caucasian population, the frequency of the G allele ranged from 4 to 36%, from 23 to 41% in Asian populations, and from 8 to 40% in mixed populations. In the control group, the frequency of the G allele varied from 10.1% to 23% (mean = 16%) (*p* = 0.5).

**Table 5:** Results of meta-analysis for the CYP1A1 gene A2455G polymorphisms and ALL risk according to the age

**Model**	**Age**	**Number of studies**	**Test of association**			**Test of heterogeneity**			**Publication bias**
			**OR**	**95% CI**	***p*-val**	**Model**	***p*-val**	**I**^**2**^	***p*-val (Egger's test)**
**Allelecontrast** **(G vs A)**	Overall	12	1,25	[1.09; 1.42]	0,00*	Fixed	0,14	30,92	0,76
	Child	10	1,18	[1.02; 1.37]	0,02*	Fixed	0,21	24,87	0,93
	Adult	1	1,75	[1.22; 2.51]	0,00*	Fixed	NA	NA	NA
	Mixt	1	1,25	[0.76; 2.06]	0,37	Fixed	NA	NA	NA
**Recessive model** **(GG vs GA+AA)**	Overall	11	1,94	[1.39; 2.71]	0,00*	Fixed	0,79	0	0,58
	Child	9	1,84	[1.27; 2.67]	0,00*	Fixed	0,82	0	0,90
	Adult	1	3,08	[1.32; 7.20]	0,00*	Fixed	NA	NA	NA
	Mixt	1	1	[0.19; 5.07]	1	Fixed	NA	A	NA
**Dominant model** ** (GG+GA vs AA)**	Overall	12	1,19	[1.01; 1.39]	0,03*	Fixed	0,19	25,08	0,77
	Child	10	1,11	[0.93; 1.32]	0,23	Fixed	0,28	17,4	0,65
	Adult	1	1,82	[1.12; 2.97]	0,01*	Fixed	NA	NA	NA
	Mixt	1	1,36	[0.75; 2.43]	0,30	Fixed	NA	NA	NA
**Homozygous** **(GG vs AA)**	Overall	11	2,02	[1.43; 2.85]	0,00*	Fixed	0,69	0	0,65
	Child	9	1,85	[1.26; 2.71]	0,00*	Fixed	0,80	0	0,97
	Adult	1	3,87	[1.59; 9.41]	0,00*	Fixed	NA	NA	NA
	Mixt	1	1,11	[0.21; 5.74]	0,89	Fixed	NA	NA	NA
**Heterozygous** **(GA vs AA)**	Overall	12	1,09	[0.92; 1.29]	0,30	Fixed	0,31	13,5	0,54
	Child	10	1,01	[0.84; 1.22]	0,84	Fixed	0,37	7,55	0,42
	Adult	1	1,55	[0.93; 2.57]	0,09	Fixed	NA	NA	NA
	Mixt	1	1,38	[0.76; 2.52]	0,28	Fixed	NA	NA	NA

*NA* Not available, *OR* Odds ratio

*significance (*p* < 0.05)

### Meta-analysis results

The results of the meta-analysis of association studies are summarized in Tables 2 and 4. By examining the heterogeneity test results, we considered both fixed and random effects models, depending on the *p*-value and I^2^ metrics. The latter was selected for subsequent analyses.

#### T3801C variant of CYP1A1 and risk of ALL

**Fig. 2:**
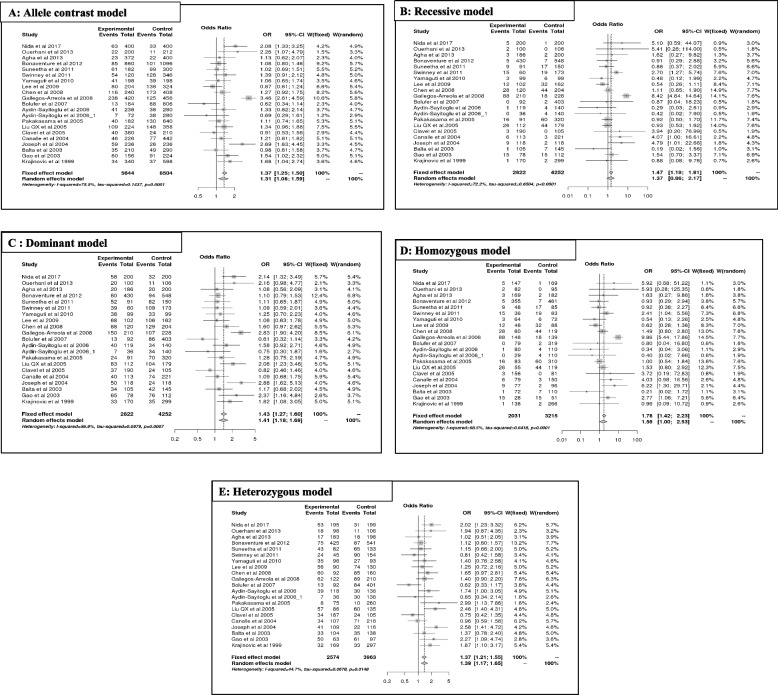
Forest plot of C3801T Polymorphism and ALL Risk under Different Models (**A**-**E**)

**Fig. 3:**
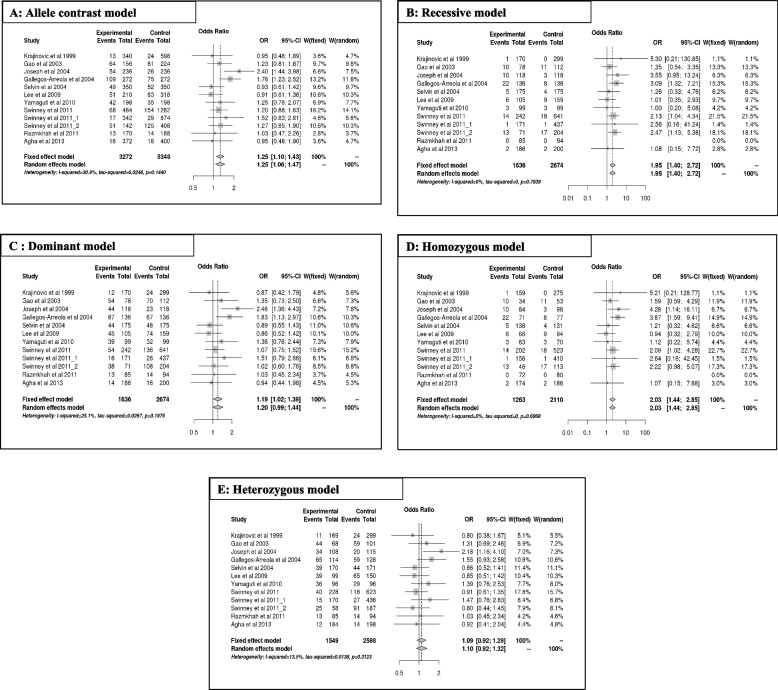
Forest plot of A2455G Polymorphism and ALL Risk under Different Models (**A**-**E**)

The results of the meta-analysis of T3801C are summarized in Table 2(A, B) and Fig. 3. Overall, the variant T3801C predicts a significant association with ALL risk, mainly in allele contrast [C vs T] (OR = 1.31, 95% CI [1.08;1.58], *p* = 0.01; Fig. 2A), dominant [CC + CT vs TT] (OR = 1.40, 95% CI [1.17;1.68], *p* = 0.00; Fig. 2C), homozygous [CC vs TT] (OR = 1.59, 95% CI [0.99;2.35], *p* = 0.05; Fig. 2D), and heterozygous models [CT vs TT] (OR = 1.38, 95% CI [1.17;1.64], *p* = 0.00; Fig. 2E), except for the recessive model [CC vs CT + TT] (OR = 1.36, 95% CI [0.86;2.16], *p* = 0.18; Fig. 2A).

According to ethnicity, the increased risk of ALL (OR > 1) was significant (*p* < 0.05), mainly in Asian populations for allele contrast, dominant, and heterozygous models, and in Hispanic populations for the homozygous model. However, susceptibility to ALL was not significant (*p* > 0.05) for other ethnicities (African and mixed). A decreased risk of ALL was observed in the Caucasian population in the recessive (OR = 0.63; 95% CI [0.30, 1.33]; *p* = 0.23) and homozygous models (OR = 0.67; 95% CI [0.32, 1.41]; *p* = 0.29). However, no risk of ALL (OR ~ 1) was observed in the Asian population in the recessive model (OR = 1.01; 95% CI [0.78, 1.31]; *p* = 0.92) or in the mixed population in the heterozygous model (OR = 1; 95% CI [0.73, 1.37]; *p* = 0.99).

Moreover, the T3801C polymorphism increased the risk of ALL, independent of age (OR > 1). As indicated by genetic models, this association was significant in children for allele contrast [C vs T] (OR = 1.27; 95% CI [1.08; 1.49]; *p* = 0.00), dominant [CC + CT vs TT] (OR = 1.35; 95% CI [1.12; 1.62]; *p* = 0.00), and heterozygous models [CT vs TT] (OR = 1.38; 95% CI [1.12; 1.69]; *p* = 0.00) and in the adult population for dominant [CC + CT vs TT] (OR = 1.85; 95% CI [1.00; 3.41]; *p* = 0.04) and heterozygous models [CT vs TT] (OR = 1.59; 95% CI [1.14; 2.21]; *p* = 0.00).

#### A2455G variant of CYP1A1 and risk of ALL

The results of the meta-analysis for A2455G are summarized in Table 4(A, B) and Fig. 3. Overall, the A2455G variant predicts a significantly increased risk of ALL, mainly in the allele contrast [G vs A] (OR = 1.25, 95% CI [1.09;1.42], *p* = 0.00; Fig. 3A), recessive [GG vs GA + AA] (OR = 1.94, 95% CI [1.39;2.71], *p* = 0.00; Fig. 3B), dominant [GG + GA vs AA] (OR = 1.19, 95% CI [1.01;1.39], *p* = 0.03; Fig. 3C), and homozygous [GG vs AA] (OR = 2.02, 95% CI [1.43;2.85], *p* = 0.00; Fig. 3D), except for the heterozygous model [GA vs AA] (OR = 1.09, 95% CI [0.92;1.29], *p* = 0.3; Fig. 3E). Regarding ethnicity, the increased risk of ALL (OR > 1) was significant mainly in mixed populations for all genetic model alleles, except for the heterozygous (OR = 1.03; 95% CI [0.830;1.32]; *p* = 0.79) and dominant models (OR = 1.15; 95% CI [0.91;1.46]; *p* = 0.23). However, no risk of ALL (OR ~ 1) was observed in Caucasian and mixed populations in the heterozygous model (OR = 1.05; 95% CI [0.78;1.42]; *p* = 0.72) and (OR = 1.03; 95% CI [0.830;1.32]; *p* = 0.79), respectively.

In addition, the A2455G polymorphism increased the risk of ALL independent of age (OR > 1). According to genetic models, this association was significant in both children and adults for allele contrast [G vs A], with respective values of (OR = 1.18 and 1.75; 95% CI [1.02; 1.37], *p* = 0.02 and 95% CI [1.22; 2.51], *p* = 0.00), recessive [GG vs GA + AA], with respective values of (OR = 1.84 and 3.08; 95% CI [1.27; 2.67]; *p* = 0.00 and 95% CI [1.32; 7.20], *p* = 0.00), and homozygous model, with respective values of (OR = 1.85 and 3.87; 95% CI [1.26; 2.71]; *p* = 0.00 and 95% CI [1.59; 9.41], *p* = 0.00). The significant association was observed only in the adult model for the dominant model (OR = 1.8, 95% CI [1.12; 2.97]; *p* = 0.01).

### Sensitivity analysis

We conducted a sensitivity analysis to evaluate the stability of the pooled results (Figs. S1 and S2). The ORs remained largely unchanged even when each individual study was excluded. This indicates that the results obtained from the meta-analysis models employed in this study are robust. Sensitivity plots for the T3801C and A2455G polymorphisms in all models are presented in Figs. S1(A-E) and S2(A-E), respectively. Studies not in HWE were excluded, as no significant changes were observed in the combined ORs.

### Publication bias

To assess publication bias, we utilized funnel plots (Figs. S3 and S4). According to the Egger’s test, the funnel plots for correlations between the T3801C and A2455G polymorphisms and the risk of ALL were symmetric, suggesting an absence of significant publication bias, except for a symmetric shape in the funnel plots. The Egger test further confirmed the absence of publication bias (all *p* > 0.05) for T3801C (Fig. S3) and A2455G (Fig. S4). However, the T3801C variant exhibited publication bias only in the adult population under the dominant model (CC + CT vs. TT).

## Discussion

In this meta-analysis, we investigated the association between CYP1A1 polymorphisms and ALL patients. This study is one of the largest across various ethnicities, age groups, and genetic models when compared to previous meta-analyses [12, 38]. Our meta-analysis included 2822 cases (4252 controls) for the C3801T variant and 1636 cases (2674 controls) for the A2455G variant of *CYP1A1*, respectively. Globally, the results of the present meta-analysis revealed that the genotypic frequencies of *CYP1A1* differed between cases and controls, although the difference was not statistically significant (*p* > 0.05). Interestingly, the highest frequency was recorded for the T3801C variant (20.6% and 25% in cases and controls, respectively). The A2455G variant was observed in 16.3% of cases and 19.5% of controls. Moreover, the frequency varied across ethnicities, reflecting the worldwide distribution of *CYP1A1* variants based on ethnicity and age [12].

Our comprehensive analysis, derived from studies to date, highlights the impact of *CYP1A1* polymorphisms on the risk of ALL, stratified by genetic models, ethnicity, and age. Overall, these variants predict an increased risk of ALL in Asian and Hispanic populations, independent of age. The T3801C polymorphism of *CYP1A1* significantly increases the risk of ALL, particularly in Asian and Hispanic populations, irrespective of age. Similarly, the A2455G polymorphism of *CYP1A1* plays a significant role in the susceptibility to ALL in all genetic models, excluding the heterozygous form. This association was predominantly observed in a mixed population of children and adults, except for the heterozygous model. These findings diverge from those reported in a previous meta-analysis by Lu et al. [12].

Nevertheless, numerous studies have indicated an association between *CYP1A1* polymorphisms and ALL, yielding inconclusive results [11–14]. The disparity in these outcomes underscores the potential impact of ethnic differences in genetic background and environmental factors related to *CYP1A1* polymorphisms and the risk of ALL. The confluence of heightened genetic susceptibility and increased environmental exposure in vulnerable populations may uniquely elevate the susceptibility to malignancy in Hispanic and Asian populations. However, investigating potential single nucleotide polymorphism–single nucleotide polymorphism interactions between *CYP1A1* and other genes across different ethnic groups could provide insights into this issue.

The polymorphism of *CYP1A1* has been reported to alter susceptibility to various types of cancers, including lung, head, neck, bladder, and breast cancer [39–41]. This discovery prompted us to explore the association between ALL and *CYP1A1* polymorphisms. In this updated meta-analysis, we present evidence suggesting that both common variants of *CYP1A1* may serve as biomarkers for ALL susceptibility. However, there are some limitations to our study. Firstly, the relatively small sample size resulted in a lack of consistently robust statistical power. Secondly, interstudy heterogeneity persists in this meta-analysis, although we minimized the likelihood through a comprehensive literature search using stringent inclusion and exclusion criteria. Finally, other factors, such as gene-gene and gene-environment interactions, should be considered. Therefore, further rigorous analyses with larger sample sizes are necessary to validate the findings of this meta-analysis.

## Conclusion

Our comprehensive analysis of studies to date indicates that *CYP1A1* variants play a role in the risk of ALL. Interestingly, both variants of *CYP1A1* (T3801 and A2455G) significantly increase the risk of ALL, depending on ethnicity. Consequently, both variants should be considered promising biomarkers for ALL risk. However, while this meta-analysis is one of the largest studies across ethnicity, age, and genetic models, further large-scale investigations are necessary to assess other factors, such as gene-gene or gene-environment interactions.

### Supplementary Information

**Additional file 1: Figure S1.** Sensitivity plot of the T3801C polymorphism under different models. A: allele contrst model, B: recessive model, C: dominant model, D: homozygous model, E: heterozygous model. **Figure S2.** Sensitivity plot of the A2455G polymorphism under different models. A: Allele contrast model, B: recessive model, C: dominant model, D: homozygous model, E: heterozygous model. **Figure S3.** Funnel plots for different models of T3801C polymorphism. A: Allele contrast model; B: Recessive model; C: Dominant model; D: Homozygous model; E: Heterozygous model. **Figure S4.** Funnel plots for different models of A2455G polymorphism. A: Allele contrast model; B: Recessive model; C: Dominant model; D: Homozygous model; E: Heterozygous model.
