# Supplementary material for: Impact of CYP1A1 variants on the risk of acute lymphoblastic leukemia: evidence from an updated meta-analysis
Source: Blood Res. 2024 Mar 4;59(1):9. doi: 10.1007/s44313-024-00007-9 (PMC10917727; doi:10.1007/s44313-024-00007-9)
Supplement: Supplementary file 1 — Additional file 1: Figure S1. Sensitivity plot of the T3801C polymorphism under different models. A: allele contrst model, B: recessive model, C: dominant model, D: homozygous model, E: heterozygous model. Figure S2. Sensitivity plot of the A2455G polymorphism under different models. A: Allele contrast model, B: recessive model, C: dominant model, D: homozygous model, E: heterozygous model. Figure S3. Funnel plots for different models of T3801C polymorphism. A: Allele contrast model; B: Recessive model; C: Dominant model; D: Homozygous model; E: Heterozygous model. Figure S4. Funnel plots for different models of A2455G polymorphism. A: Allele contrast model; B: Recessive model; C: Dominant model; D: Homozygous model; E: Heterozygous model. [file 44313_2024_7_MOESM1_ESM.docx]

Supplement

| 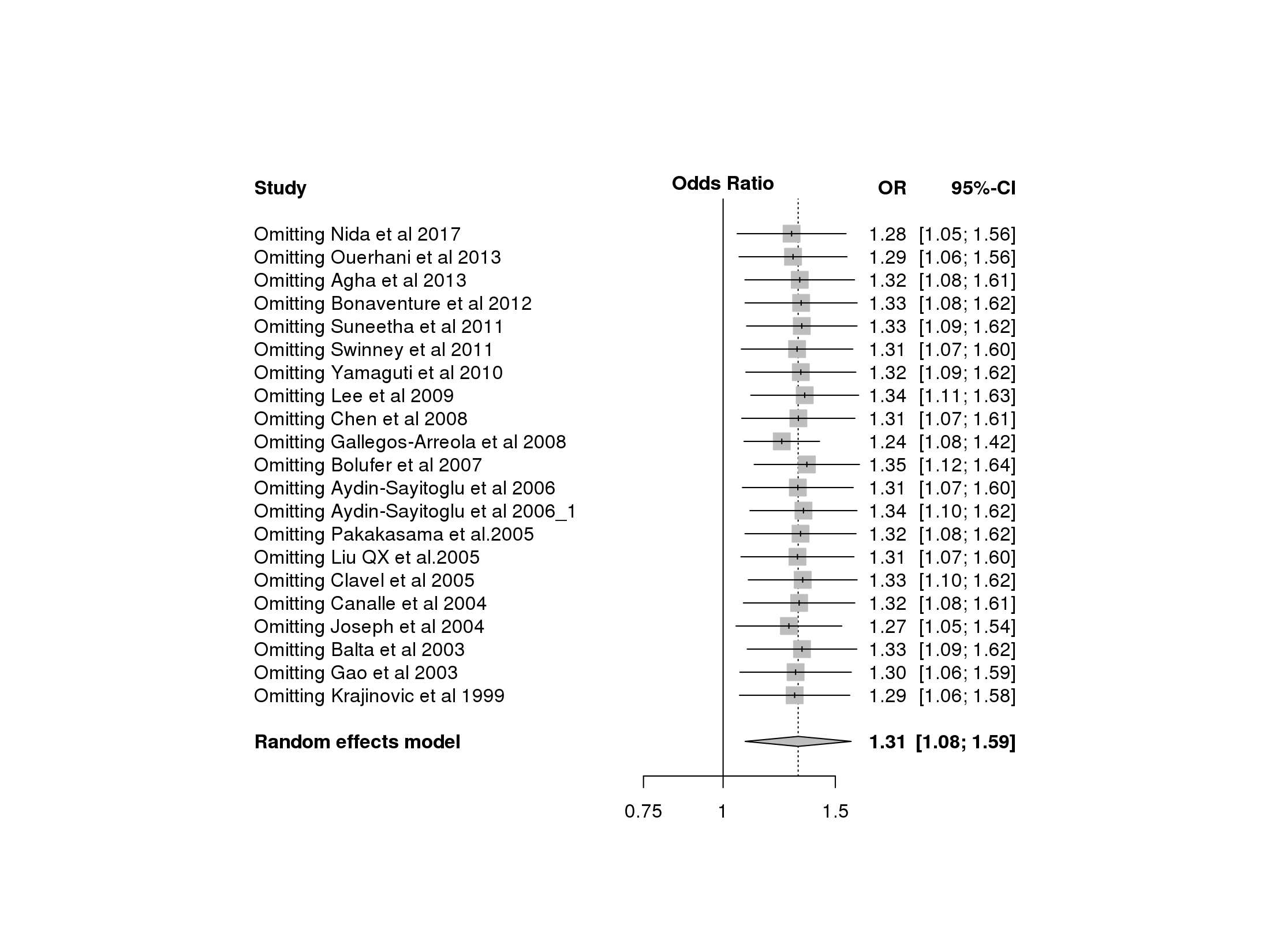  **A** | 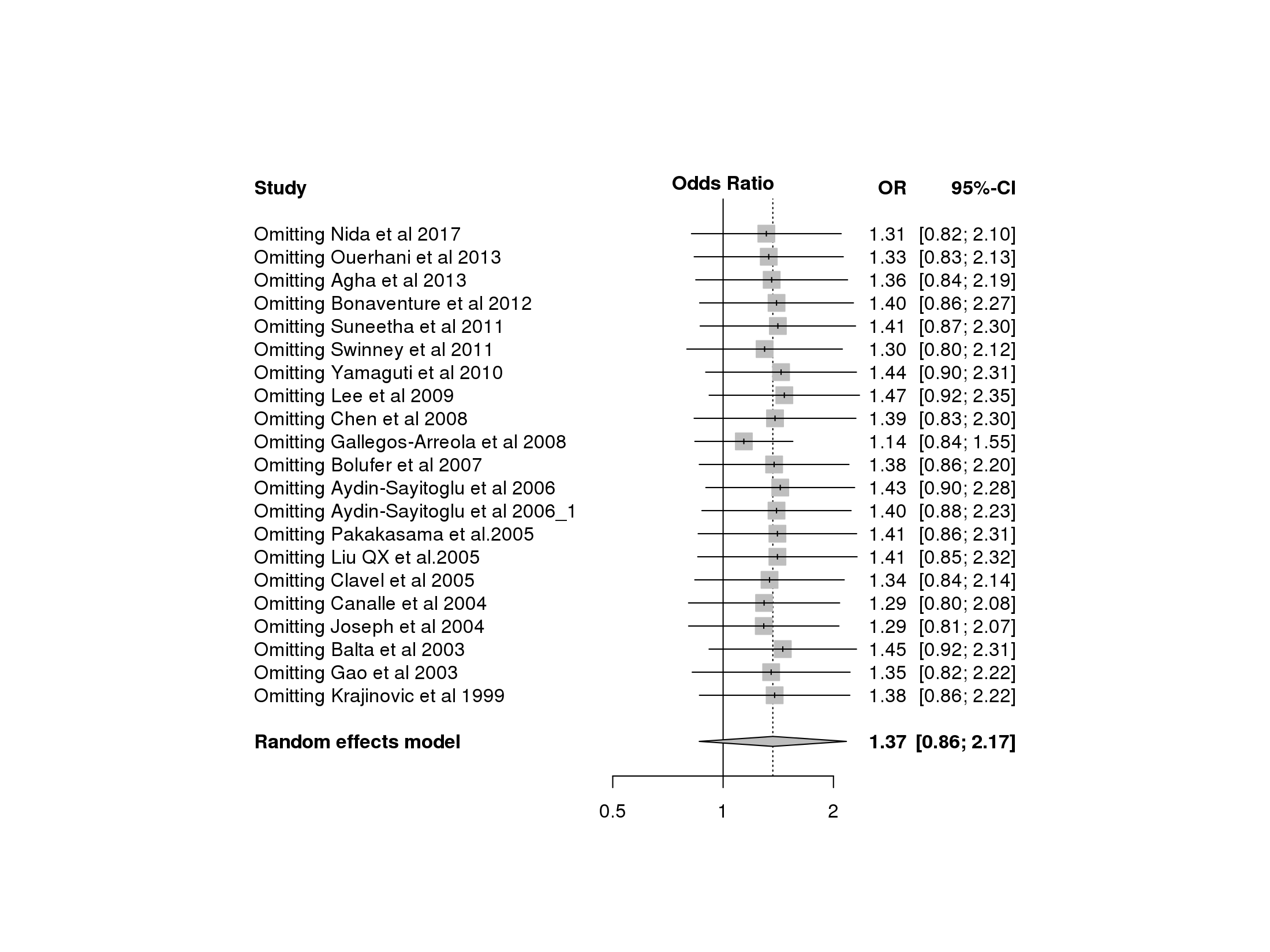  **B** | 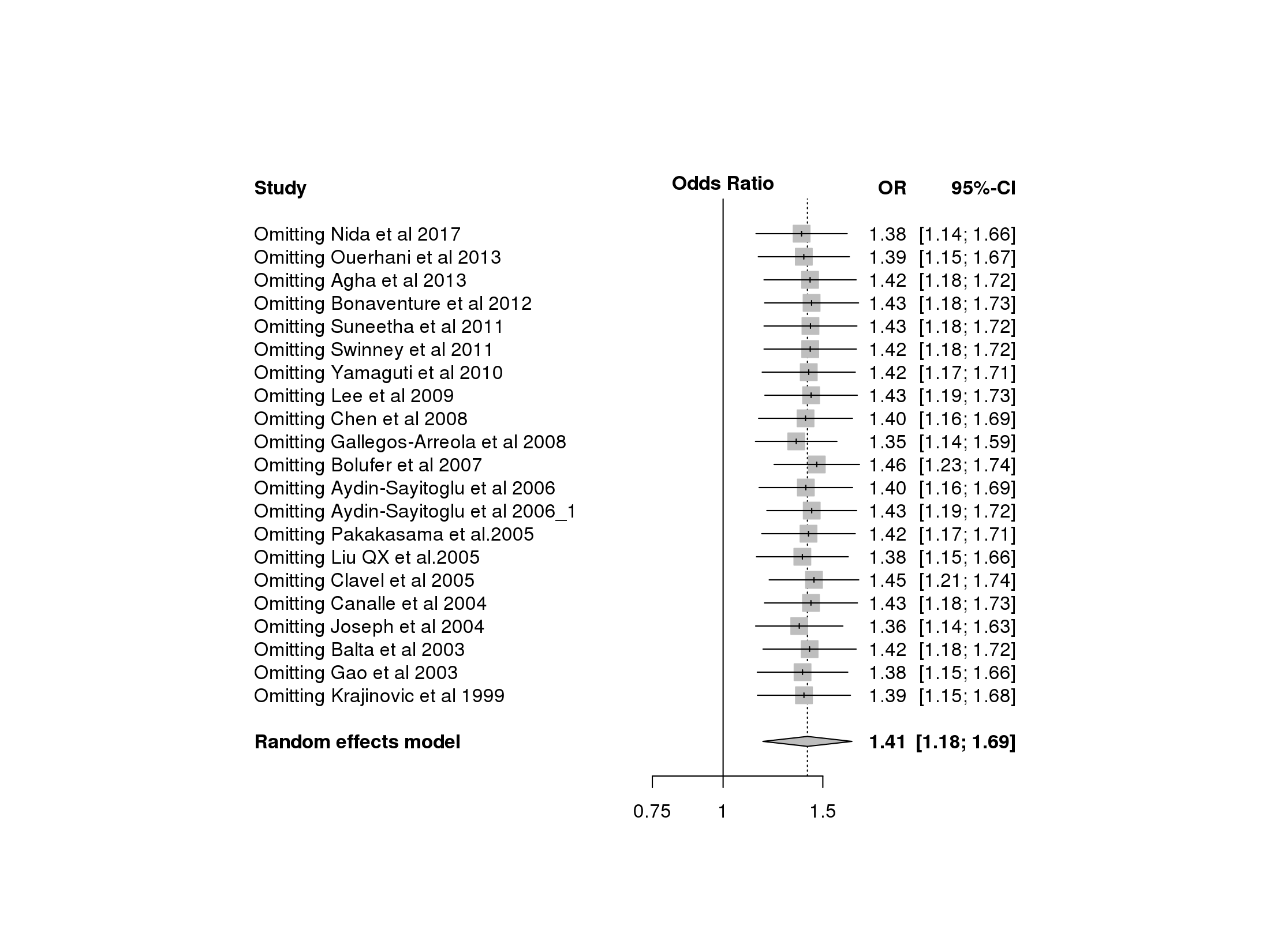  **C** |
| --- | --- | --- |
| 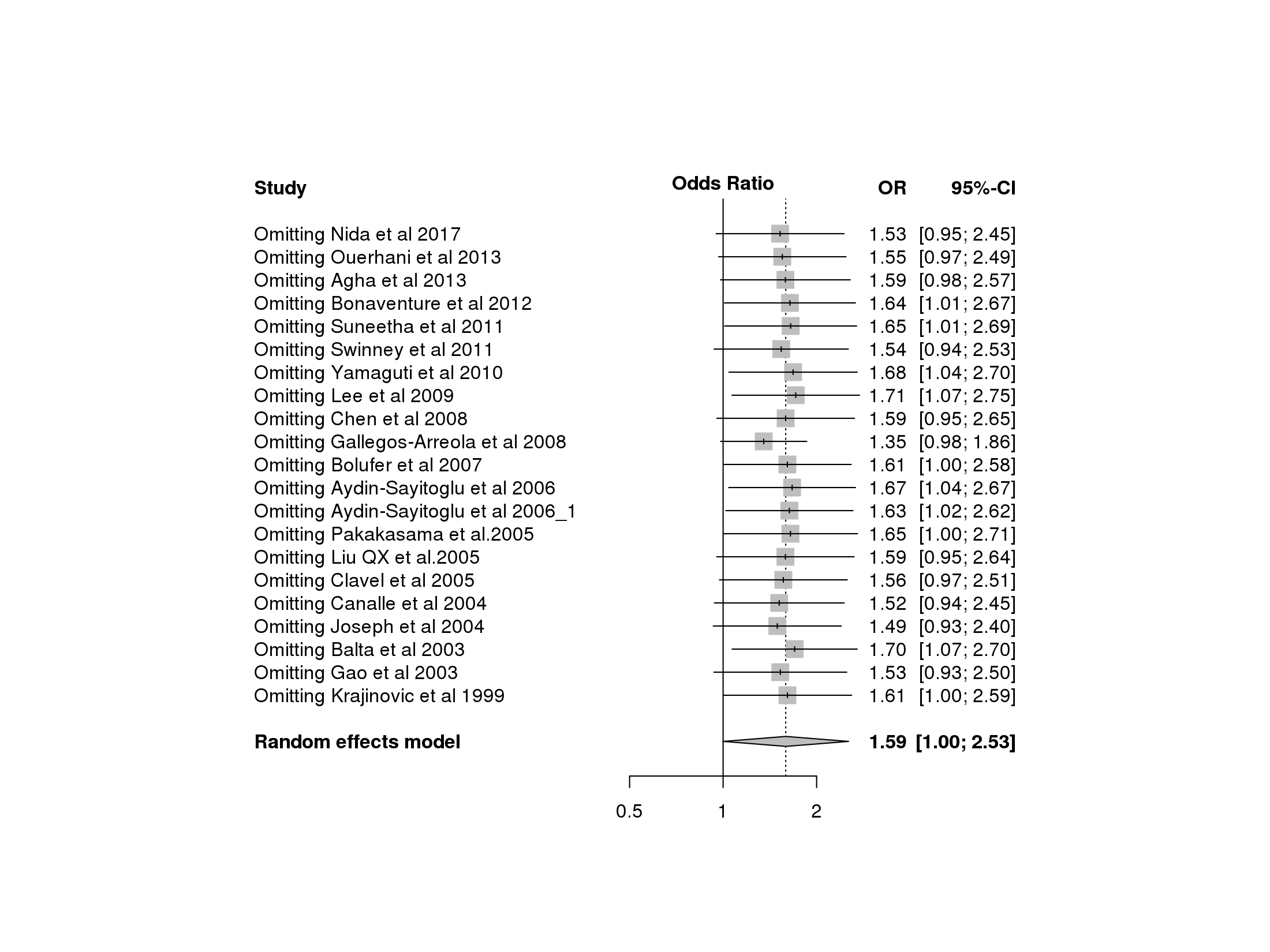  **D** | 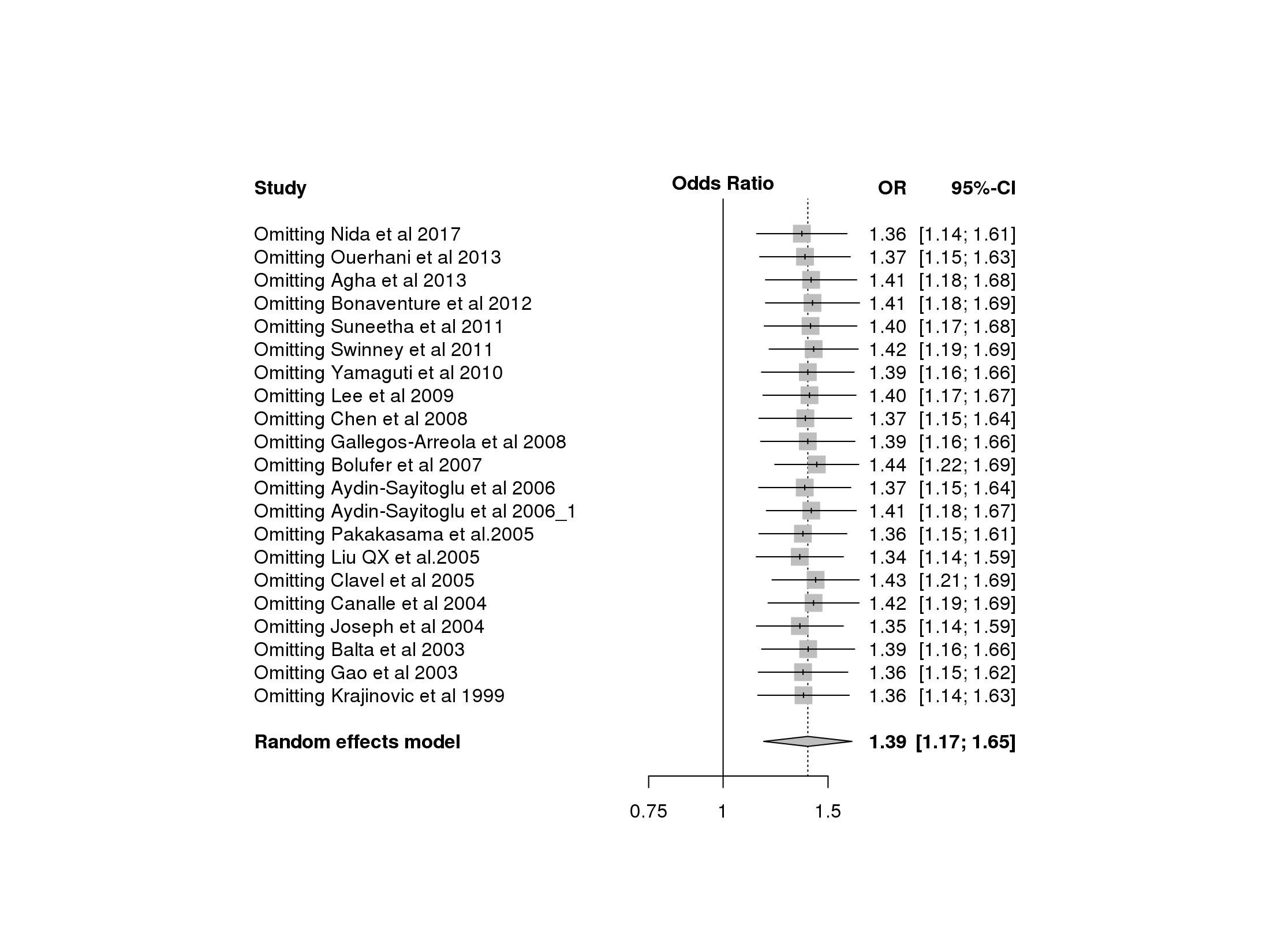  **E** | Figure S1: Sensitivity plot of the T3801C polymorphism under different models.  A : allele contrst model, B : recessive model, C : dominant model, D : homozygous model, E : heterozygous model. |

| 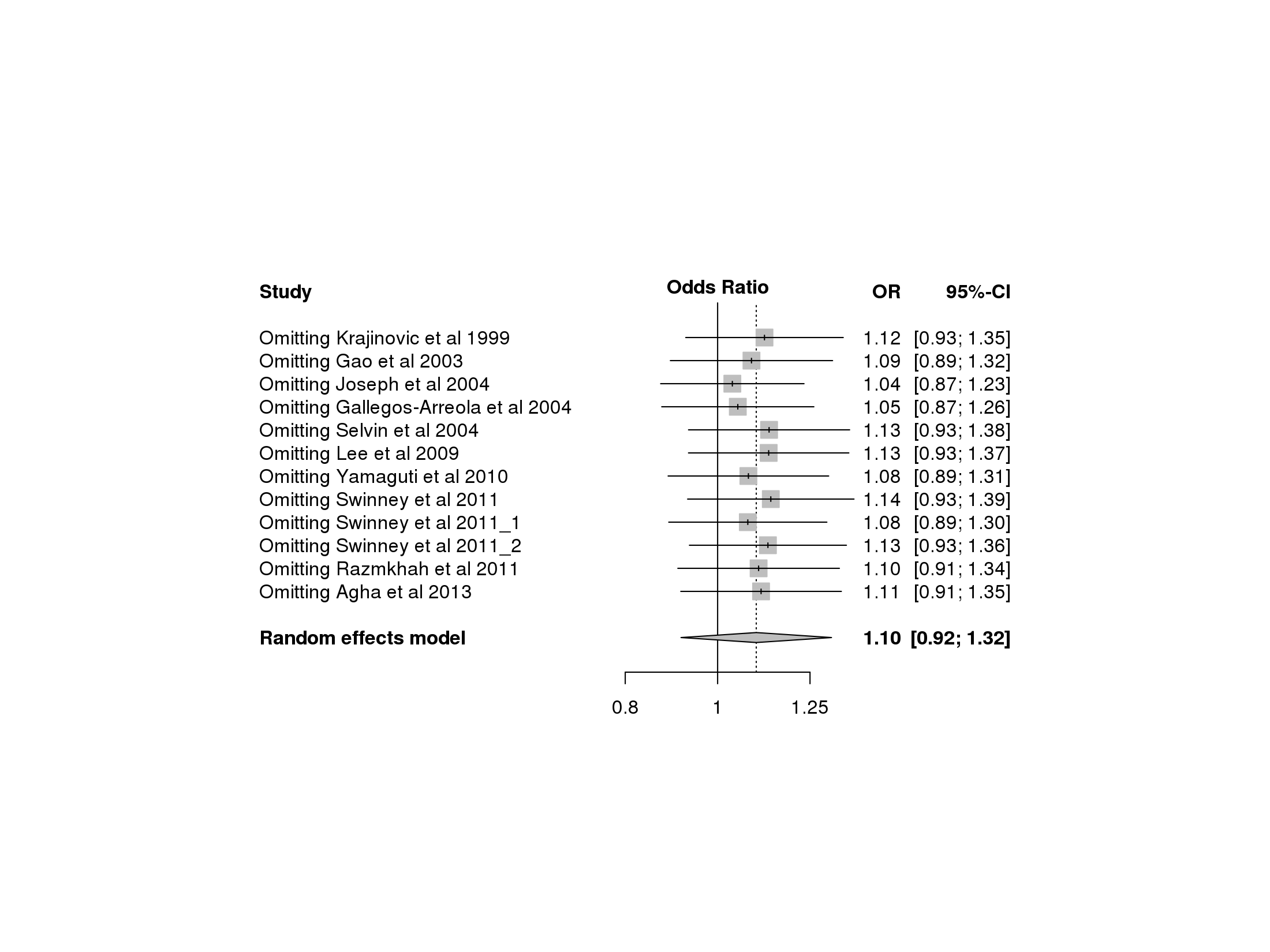  **A** | 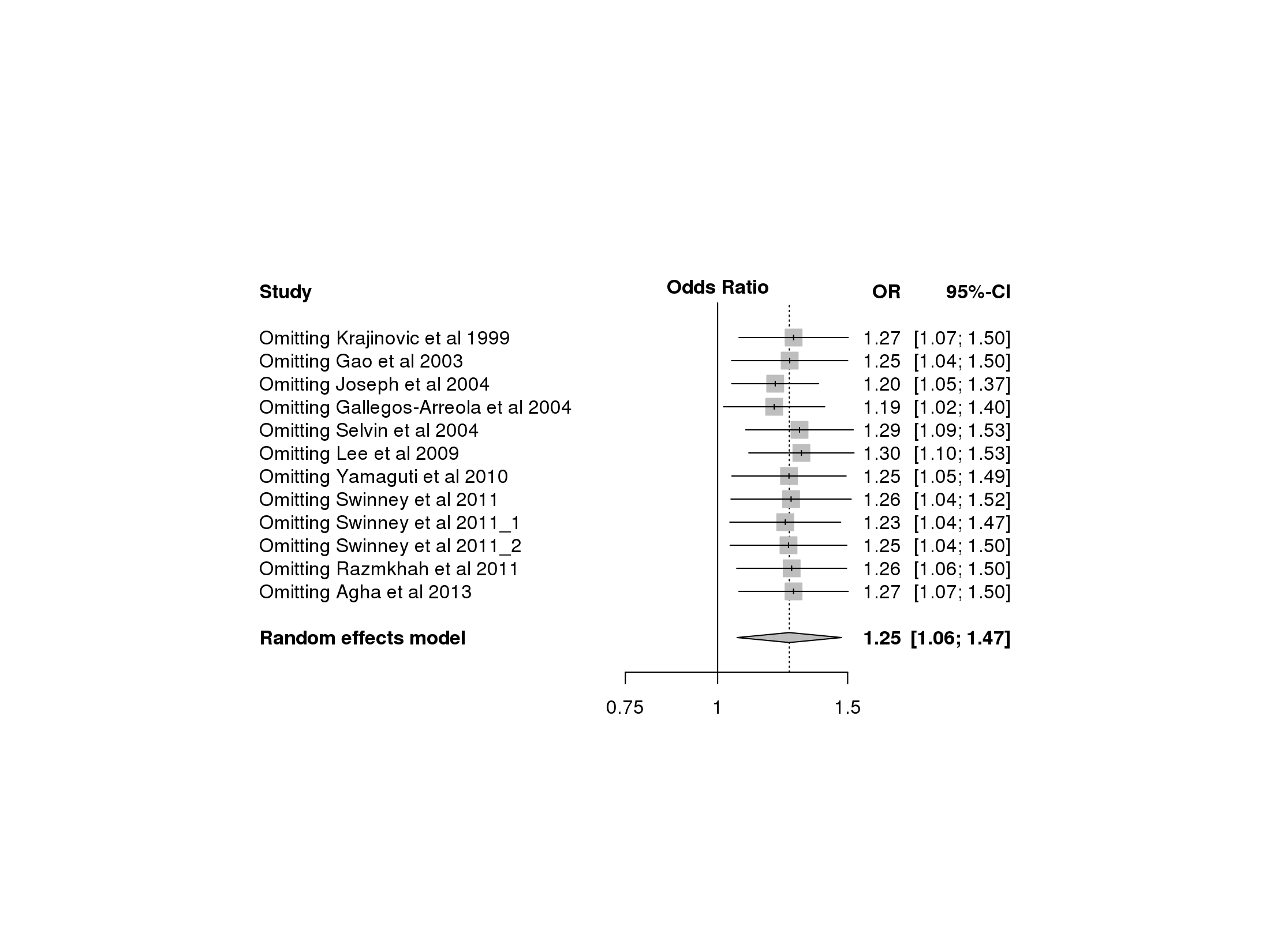  **B** | 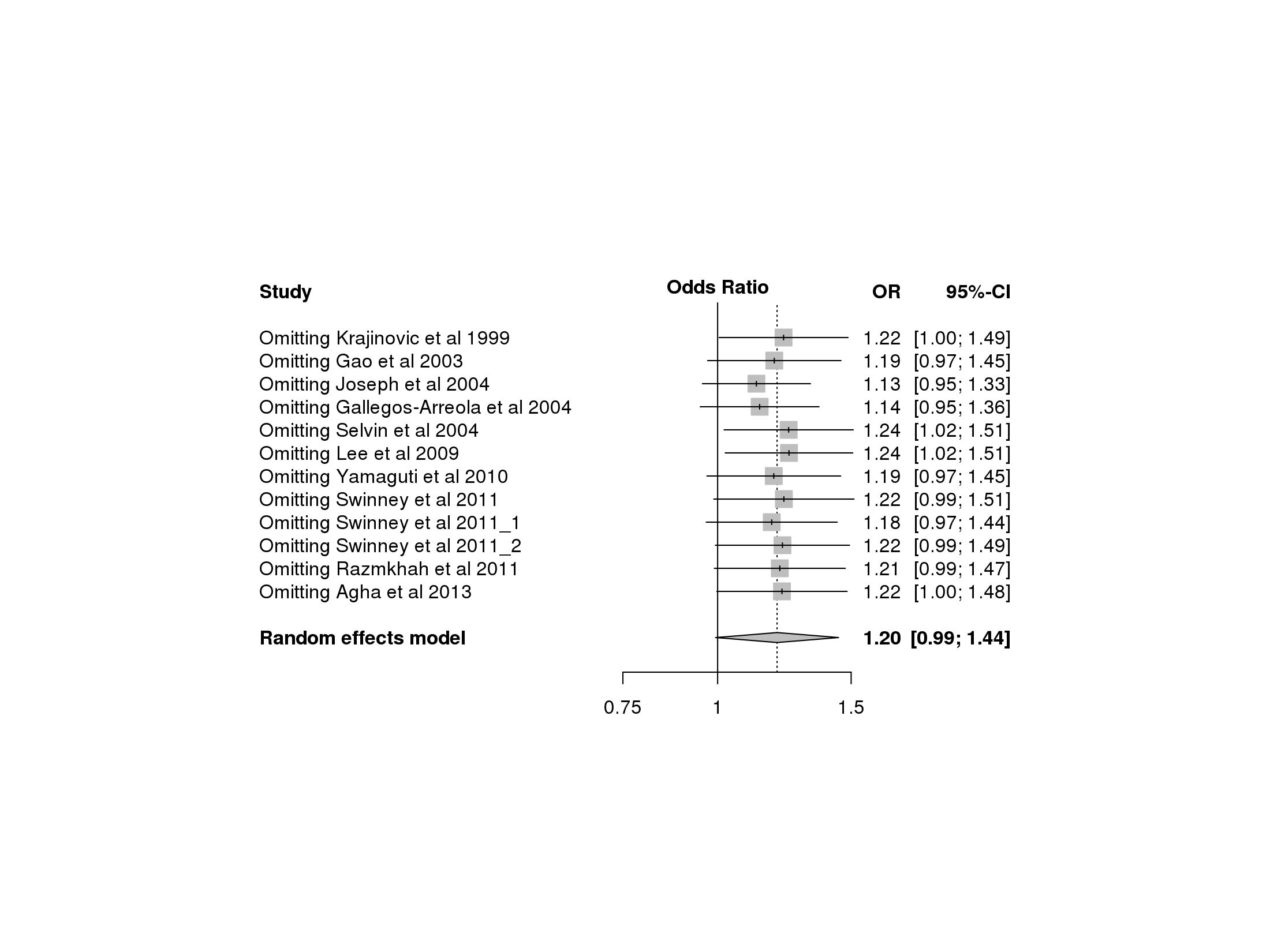  Figure S2: Sensitivity plot of the A2455G polymorphism under different models.  A : Allele contrast model, B : recessive model, C : dominant model, D : homozygous model, E : heterozygous model.  **C** |
| --- | --- | --- |
| 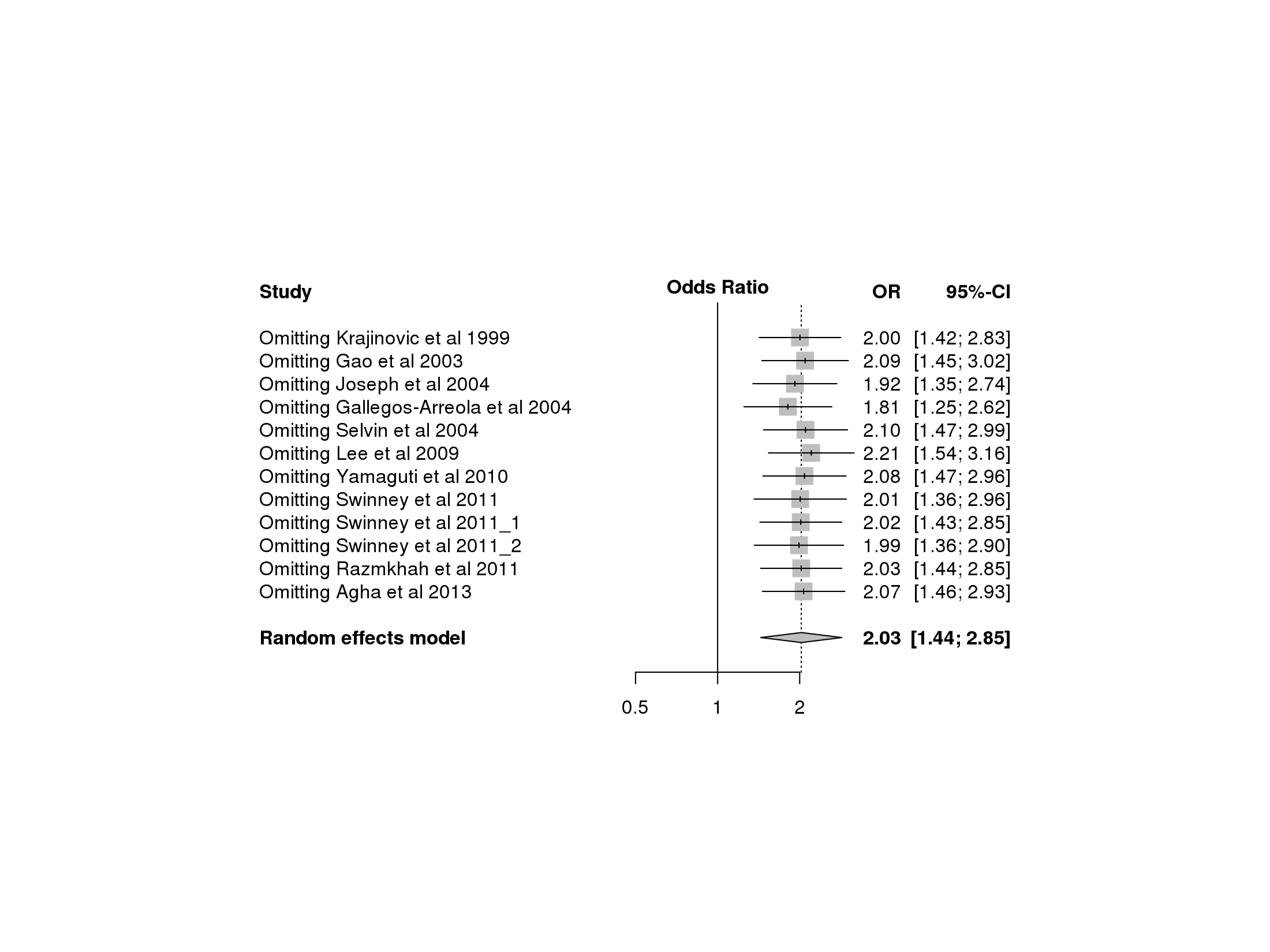  **D** | 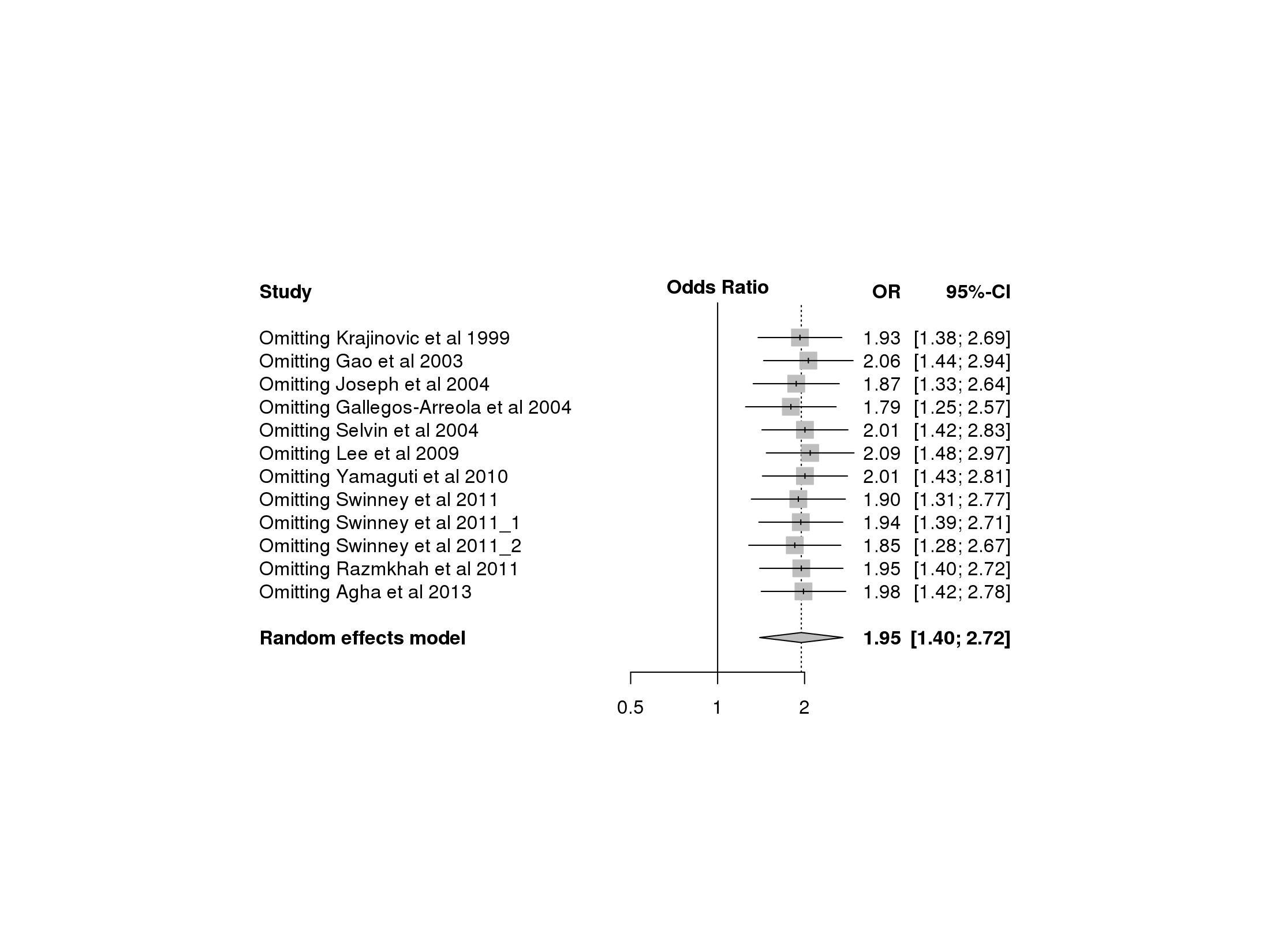  **E** |  |


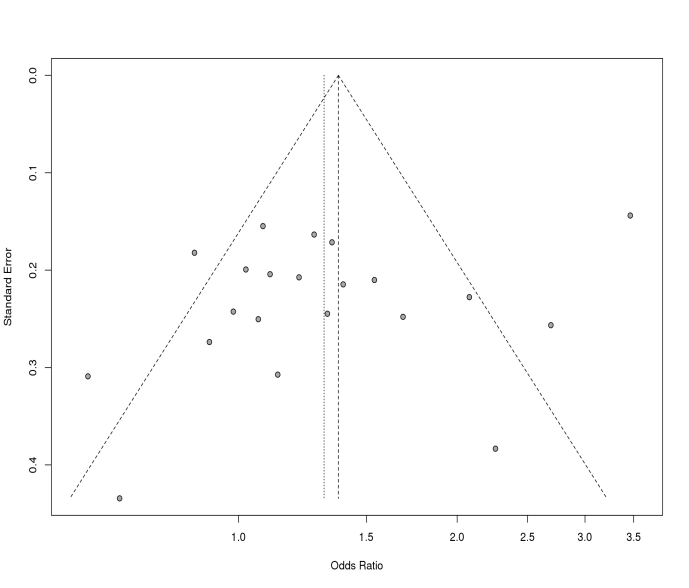

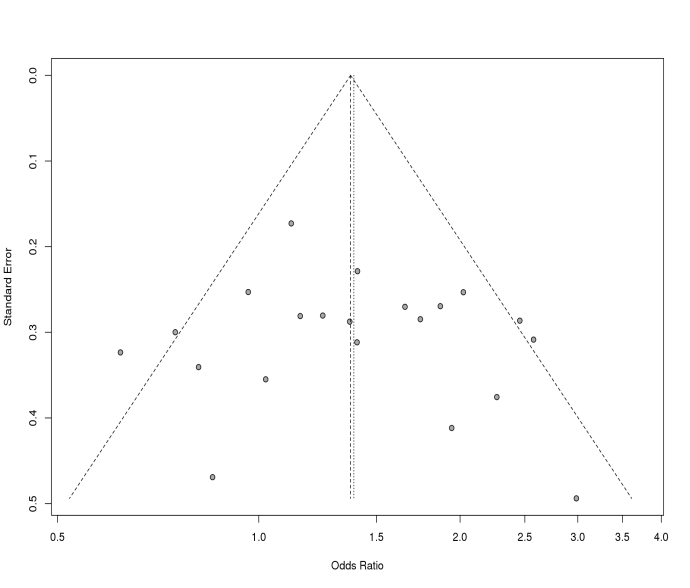

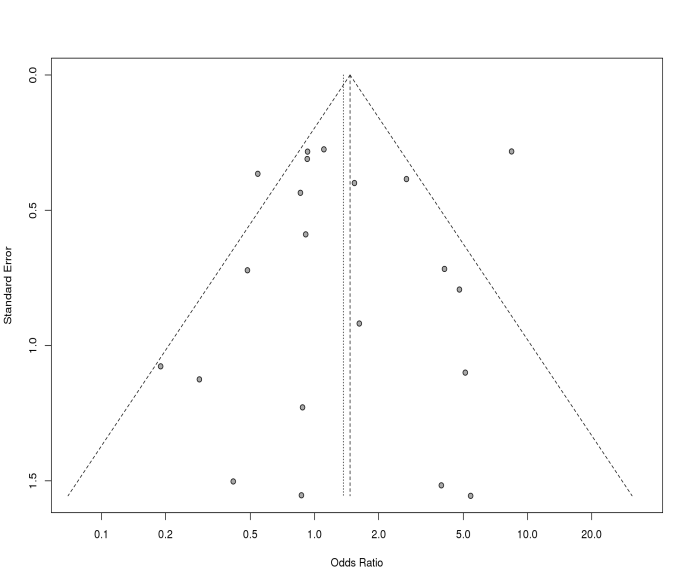


**C**

**A**

**B**


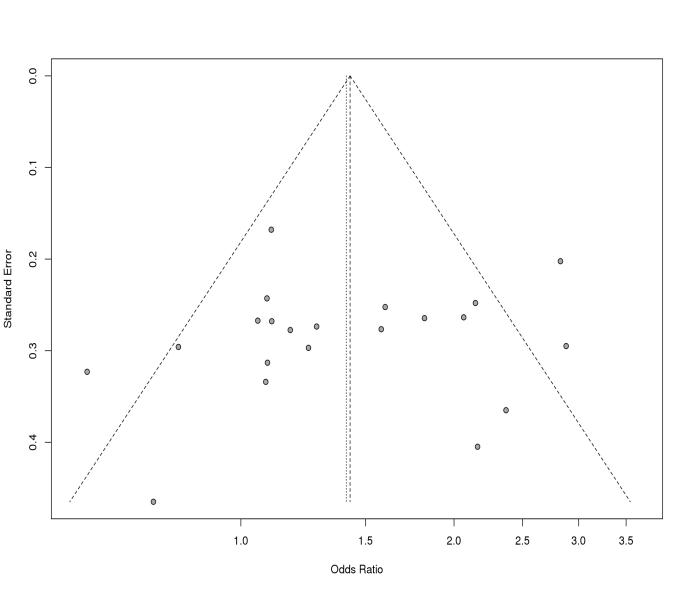

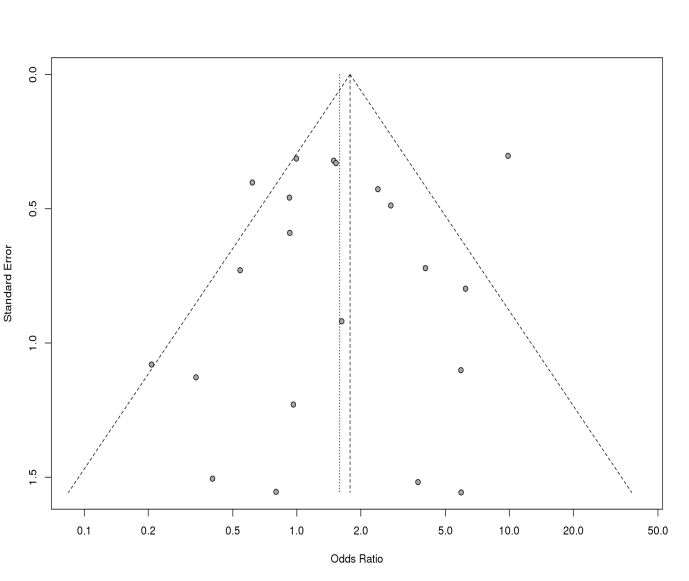


**E**

**D**

**Figure S3: Funnel plots for different models of T3801C polymorphism**

A : Allele contrast model ; B : Recessive model ; C : Dominant model ; D : Homozygous model ; E : Heterozygous model


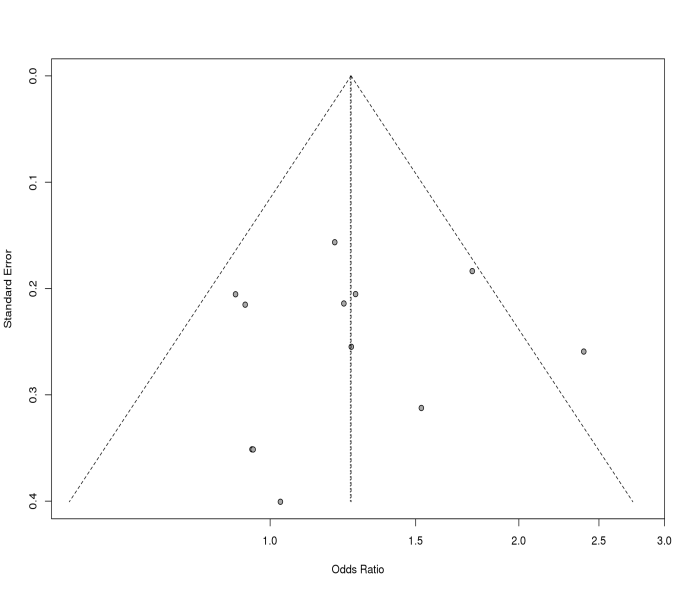

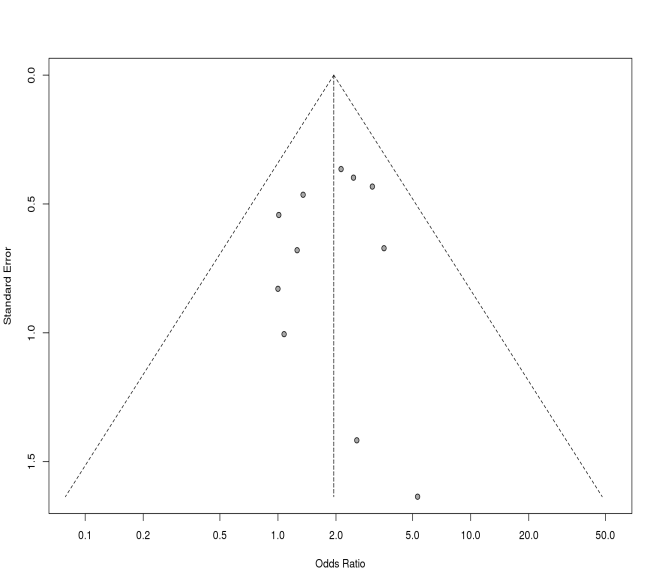

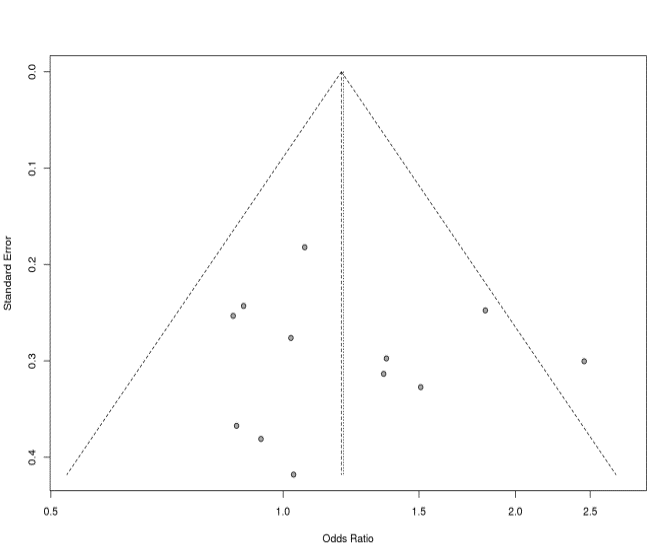


**Figure S4: Funnel plots for different models of A2455G polymorphism**

A : Allele contrast model ; B : Recessive model ; C : Dominant model ; D : Homozygous model ; E : Heterozygous model

**C**

**B**

**A**


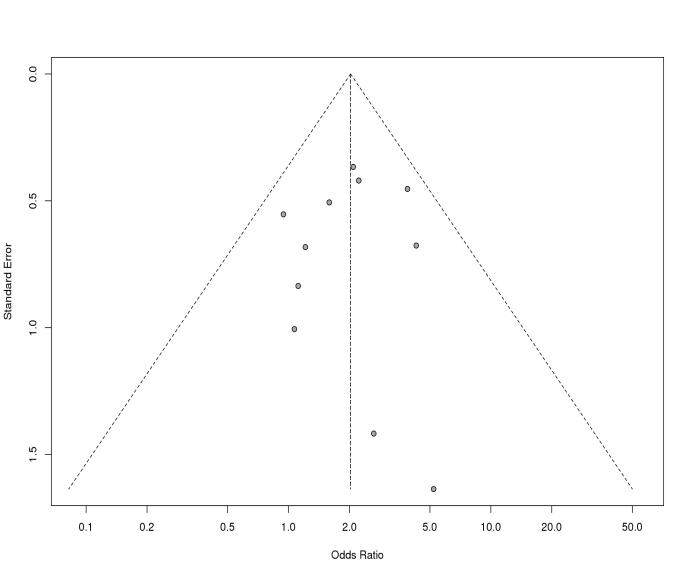

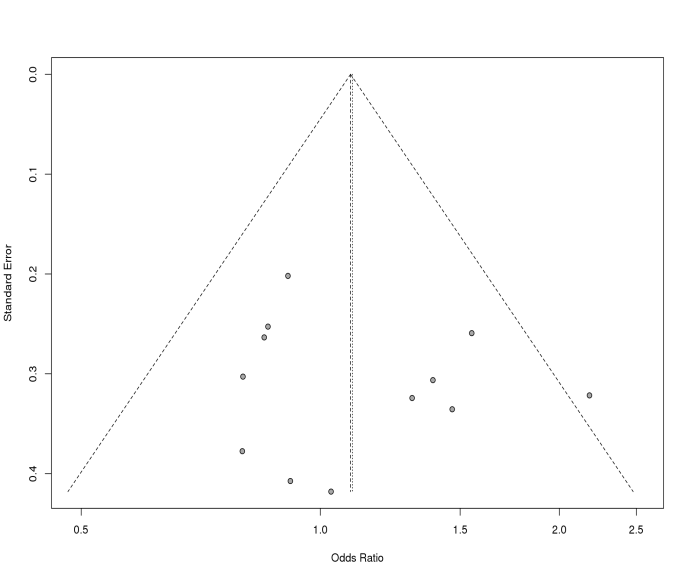


**E**

**D**
